# Diagnosis in a Preclinical Model of Bladder Pain Syndrome Using a Au/ZnO Nanorod-based SERS Substrate

**DOI:** 10.3390/nano9020224

**Published:** 2019-02-07

**Authors:** Sanghwa Lee, Jung-Man Namgoong, Hwan Yeul Yu, Miyeon Jue, Gwanho Kim, Sangmin Jeon, Dong-Myung Shin, Myung-Soo Choo, Jinmyoung Joo, Chan-Gi Pack, Jun Ki Kim

**Affiliations:** 1 Biomedical Engineering Research Center, Asan Medical Center, Seoul 05505, Korea; pause1919@gmail.com (S.L.); imascai@naver.com (M.J.); 2 Department of Surgery, Asan Medical Center, University of Ulsan College of Medicine, Seoul 05505, Korea; namgoong2940@amc.seoul.kr; 3 Department of Urology, Asan Medical Center, University of Ulsan College of Medicine, Seoul 05505, Korea; hwanyel@naver.com (H.Y.Y.); mschoo@amc.seoul.kr (M.-S.C.); 4 Department of Biomedical Sciences, Asan Medical Center, University of Ulsan College of Medicine, Seoul 05505, Korea; d0shin03@amc.seoul.kr (D.-M.S.); changipack@amc.seoul.kr (C.-G.P.); 5 Department of Chemical Engineering, Pohang University of Science and Technology, Pohang 37673, Korea; kgoanho929@postech.ac.kr (G.K.); jeons@postech.ac.kr (S.J.); 6 Department of Biomedical Engineering, Ulsan National Institute of Science and Technology, Ulsan 44919, Korea; jjoo@unist.ac.kr; 7 Department of Convergence Medicine, University of Ulsan College of Medicine, Seoul 05505, Korea

**Keywords:** interstitial cystitis/bladder pain syndrome (IC/BPS), ZnO nanorods, surface enhancement Raman spectroscopy (SERS), principal component analysis (PCA)

## Abstract

To evaluate the feasibility of ZnO nanorod-based surface enhanced Raman scattering (SERS) diagnostics for disease models, particularly for interstitial cystitis/bladder pain syndrome (IC/BPS), ZnO-based SERS sensing chips were developed and applied to an animal disease model. ZnO nanorods were grown to form nano-sized porous structures and coated with gold to facilitate size-selective biomarker detection. Raman spectra were acquired on a surface enhanced Raman substrate from the urine in a rat model of IC/BPS and analyzed using a statistical analysis method called principal component analysis (PCA). The nanorods grown after the ZnO seed deposition were 30 to 50 nm in diameter and 500 to 600 nm in length. A volume of gold corresponding to a thin film thickness of 100 nm was deposited on the grown nanorod structure. Raman spectroscopic signals were measured in the scattered region for nanometer biomarker detection to indicate IC/BPS. The Raman peaks for the control group and IC/BPS group are observed at 641, 683, 723, 873, 1002, 1030, and 1355 cm^−1^, which corresponded to various bonding types and compounds. The PCA results are plotted in 2D and 3D. The Raman signals and statistical analyses obtained from the nano-sized biomarkers of intractable inflammatory diseases demonstrate the possibility of an early diagnosis.

## 1. Introduction

Raman spectroscopy is useful for verifying the characteristics of biological samples ranging from nanoscale to millimeter size, such as tissue [1,2], cells [3,4,5], bacteria [6,7], exosomes [8,9], and proteins [10,11]. In the Raman spectroscopy of biological samples, the detection of high-performance nanometer-sized biomarkers is attracting much research interest due to its application for the early diagnosis of diseases. For highly sensitive liquid and optical biopsies, in this work, an approach to Raman signal enhancement on biosensing chips based on surface enhancement Raman spectroscopy (SERS) diagnostics was utilized. Previously, a significant signal enhancement has been realized by using a porous ZnO nanostructure for the bio-liquid sample. The clustering of Au nanoparticles on ZnO nanorods was the main factor affecting the enhancement of local surface plasmon resonance (LSPR), as demonstrated by a finite element method (FEM) analysis [12]. It is possible to utilize surface enhanced Raman chips based on such porous nanostructures to ensure advantages in applications such as measuring liquid samples, including aqueous humors [13], eliminating the effect of coffee rings, and studying living cells [12]. Furthermore, by using the property that Raman signals are enhanced at the side of the Au coated ZnO nanorods [12], and by controlling the porosity through the modification of parameters such as the length, diameter and density of ZnO nanorods [14,15], nanometer sized targets can be measured selectively.

Interstitial cystitis/bladder pain syndrome (IC/BPS) is a refractory disease that causes pelvic pain when urine enters the bladder, and also causes frequent urination [16]. It is a chronic inflammatory state of the submucosal and muscular layer of the bladder, which is characterized by urothelium denudation, mast-cell activation, and sensory nerve hyperactivation, and it is often associated with sexual dysfunction, sleep dysfunction, depression, anxiety, and chronic stress [17,18]. There are various treatments for this affliction based on oral agents [19,20,21], but they are unsatisfactory, with frequent recurrences of symptoms and of Hunner lesions [17]. Currently, other solutions, such as mesenchymal stem cell therapy, are being developed to alleviate interstitial cystitis. However, along with the development of therapeutic technology, the early diagnosis of IC/BPS can be beneficial to quality of life. Further, the detection of the disease before its development into the chronic stages can minimize the patient’s pain and increase the effectiveness of the treatment.

**Figure 1:**
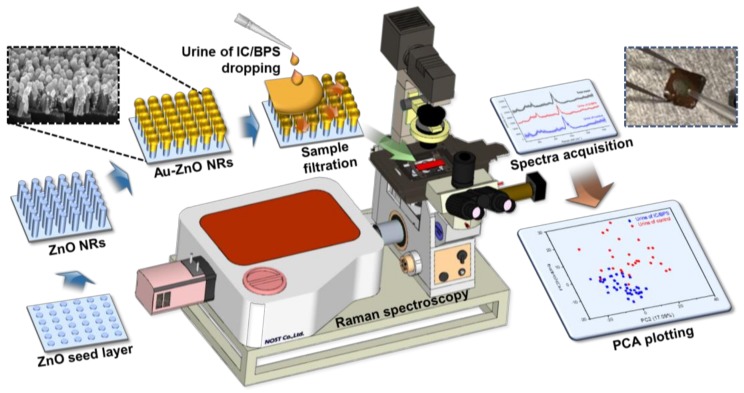
Schematic showing the production process, measurement, and analysis process of the ZnO nanorod-based SERS sensing chip.

Compared to standard Raman spectroscopy, SERS enables an early detection by exhibiting a signal enhancement of 8–10 orders of magnitude, which is formed at the nanometer gap between Au/ZnO nanorods [22,23]; therefore, selecting and targeting nanometer-sized biomarkers is key to applying SERS technology for an early diagnosis. If we develop a technique to detect nano-level biomarkers of inflammatory diseases in urine or other fluids, a noninvasive diagnosis can be realized, preventing pain and discomfort for the patient. As shown in Figure 1, in this paper, we manufactured a ZnO-based nanorod analysis chip to acquire Raman signals from the obtained urine in the IC/BPS pre-clinical animal model. Raman signals and statistical analyses obtained from nano-sized biomarkers of intractable inflammatory diseases that cause pain in patients demonstrate the possibility of an early diagnosis.

## 2. Materials and Methods

### 2.1. Urine Sampling of IC/BPS Rat Model

#### 2.1.1. IC/BPS Rat Model

Previous research has shown that an intravesical injection of hydrochloric acid (HCl) induces IC/BPS symptoms in rats [24,25]. IC/BPS animal models and comparative groups (*n* = 4 each) were derived using 10-week-old female Sprague–Dawley rats, as in a previous paper [26]. The rats were injected with 0.2 M HCl for 10 min using a 26-gauge angiocatheter in the bladders, followed by neutralization and a saline wash. Four rats in the comparison group were used as vehicles, and were not subject to an HCl injection.

#### 2.1.2. Urine Extraction and Analysis of Voiding Pattern

Rat urine was collected in a 50 mL tube using a metabolic cage. As in a previous paper [26], the voiding pattern measured a week after the HCl injection was examined, and the collected urine was used as a sample for the Raman measurement. Twenty-four hours of natural voiding patterns in the metabolic cage were recorded and analyzed using Acq Knowledge 3.8.1 software and an MP150 data acquisition system (Biopac Systems, Goleta, CA, USA), at a sampling rate of 50 Hz. The change in the urine volume for the model group, as obtained from raw data, was estimated at 0.5 mL.

### 2.2. Surface Enhanced Raman Measurements

#### 2.2.1. ZnO Nanorod Based SERS Chip

As shown on the left side of Figure 1, to amplify the Raman signal using the SERS substrates, the Si wafer was initially scribed and broken into pieces with a 1 × 1 cm^2^ size to form substrates for ZnO nanorods. It was cleaned in ethanol and de-ionized (DI) water for 5 min. The 30 nm ZnO seed layer was deposited on the surface of the as-prepared samples by using RF magnetron sputtering for 5 min under 100 W power, to grow the vertically aligned ZnO nanorods through a hydrothermal synthesis. The ZnO growth solution was prepared by dissolving 10 mM zinc nitrate hexahydrate (Sigma Aldrich Co., St. Louis, MO, USA) and 0.9 mL of ammonium hydroxide (Sigma Aldrich Co., St. Louis, MO, USA) in 30 mL of DI water. To create a homogeneous aqueous solution, it was mildly stirred using a vortexer for 5 min at room temperature. Then, the as-prepared samples were immersed in the aqueous solution in an oven at 90 °C for 50 min. After ZnO growth, the substrates were cleaned with DI water and then dried using nitrogen gas. Finally, the ZnO nanorods were coated with Au using a thermal evaporator (Alpha Plus Co., Ltd., Asan, Korea), and the coating was stopped when a thin film thickness monitor showed an equivalent thickness of 100 nm. The morphological and structural properties of the Raman measured samples were observed using a field-emission scanning electron microscope (FE-SEM) (S-4700, HITACHI, Tokyo, Japan) with a 10 kV beam voltage.

#### 2.2.2. Raman Spectra Acquisition and Analysis

A drop of the 5 µL sample was applied to the substrate; the sample had a spreading time of 60 min during which it diffused into the nanometer-scale pores between the nanorods, and was filtered as a result, before drying on the substrate. After confirming that the droplets were dry and diffused, they were loaded onto a Raman spectroscopy system attached to a microscope (IX-73, Olympus, Tokyo, Japan) and measured. Raman spectra were collected using a customized spectrometer (FEX-INV, NOST, Seongnam, Korea) with a 785 nm diode laser as the excitation source. The 1 mW of excitation light was focused on the sample through a 40×/0.6 NA objective with a measured spot size of approximately 2.4 μm, as imaged through the microscope. The spectrum of each irradiation point on the substrate from which Raman spectra were gathered was measured 8 times in the range of 550 to 1500 cm^−1^, with a spectral resolution of 1 cm^−1^ and an integration time of 40 s at room temperature. The Raman spectrum was calibrated by measuring a silicon sample before the Raman measurements. After the Raman measurements, the spectrum was postprocessed by 3rd-order polynomial fitting to remove the auto-fluorescence background and by Savitzky–Golay smoothing. To evaluate the spectral differences between the control and IC/BPS rat urine, a principal component analysis (PCA) was used. PCA reduces the number of variables in multivariate systems, so all of the spectral range was used as variables. All analyses were conducted using the XLSTAT 2018 software.

## 3. Results

### 3.1. IC/BPS Rat Models and Sample Preparation

#### 3.1.1. Voiding Frequency and Sample Drop

**Figure 2:**
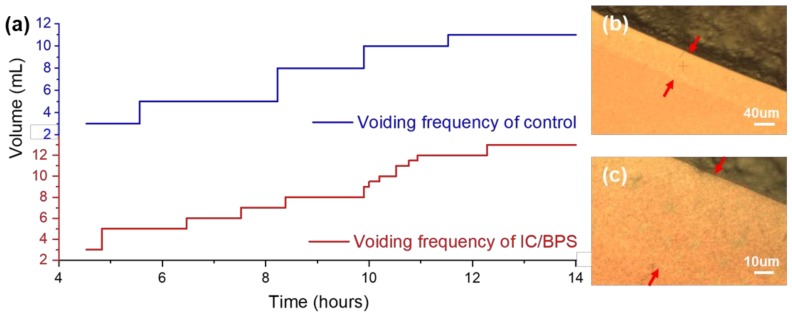
(**a**) Measurement of voiding function in the control group and the IC/BPS animal group at 7 days after HCl treatment. Optical microscope images of a Raman measurement region diffused from a sample droplet into a nanoporous area, indicated by red arrows: (**b**) 10× and (c) 40× objective.

To confirm the implementation of interstitial cystitis, the voiding pattern after a week for the control group and HCl treated rats was measured. As shown in Figure 2a, an irregular voiding frequency is observed in the HCl-treated rats, which indicates the urinary dysfunction caused by bladder inflammation, and matches that of a previous animal model experiment [17,26]. In the graph, one step and a terrace represent the volume increase and the duration between voiding, respectively. The total amount of the control group and the IC/BPS animal model for about 10 h is 11 and 13 mL, while the frequency is 4 and 11 times, respectively.

When the urine that has an abnormal voiding status is dropped on the SERS substrate, as shown in the inset on the right upper side of Figure 1, a droplet is formed due to surface tension and is dried. When the edge of the droplet is observed with an optical microscope, the band region, as shown in Figure 2b,c, is formed. A sample was soaked in this region where the nanometer gap was formed from the gold-coated ZnO nanorods. Since the urine contained a few micrometer-sized bacteria, red blood cells approximately 7 μm in diameter, epithelial cells of several tens of microns in diameter, and so on, the measurement of urine by non-SERS Raman spectroscopy from a number of microbeam irraditation spots results in a significant variation in the results. Therefore, filtering by the nano-level porosity of the nanorods facilitates the spectroscopic measurement, by eliminating micron-scale noise sources.

#### 3.1.2. Analysis of Measurement Area

**Figure 3:**
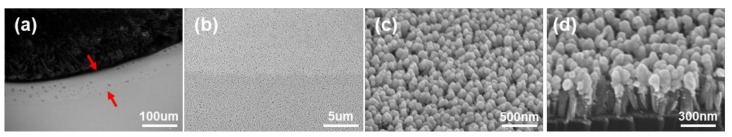
FE-SEM images of urine dropped on a SERS substrate: (**a**) 250 times magnification, containing a dried droplet, diffused area, and bare SERS area; (**b**) 5k and (**c**) 50k magnification showing the interface between the diffused and bare area; (**d**) cross-sectional image showing Au-coated ZnO nanorods.

On the electron microscope image for the Raman measurement, the validity of the points for spectral acquisition of the nanometer target was next investigated. In the electron microscope, the droplet area of the specimen is darkened, and the bare SERS area is brightly contrasted. The thickness of the urine-diffused band region, shown in Figure 2b, under an optical microscope is 60 μm, which is in agreement with the thickness of the band region indicated by the red arrow in Figure 3a. As shown in Figure 3b, the interface between the diffuse region and the bare SERS region of the urine droplet has a brightness contrast, but the magnitude of the interface contrast seems to be the same as that in Figure 3c. The difference in the boundaries shown in Figure 3b appears to be due to the difference in the secondary electron emission characteristics, whereas the resolution in Figure 3c appears to exhibit no difference in morphology. The Figure 3 images show secondary electrons emitted by the scanning electron beam and display them, and these emitted electrons are orientated on the surface. Thus, the contrast of the diffused region is different from that of the bare SERS; this is the difference in the emission electron characteristics produced by the addition of different materials to the gold, which is well below the scale of tens of nanometers.

In the SERS substrate deposition, the nanorods grown after the ZnO seed deposition were 30–50 nm in diameter and 500–600 nm in length. The image of gold deposited on the nanorod structure with a thickness of 100 nm is shown in Figure 3d. The deposited gold forms clusters at the ends of the ZnO nanorods, and these clusters have diameters in the range of 80 to 100 nanometers. A finite element method (FEM) analysis of the thickness and length of the ZnO rod and the diameter of the head was conducted in a previous study [12], and it was confirmed from the FEM results that the local surface plasmon resonance (LSPR) is formed on the side of the rod and on the head. The electric field formation of the incident electromagnetic waves is formed in a direction perpendicular to the traveling direction, which constitutes the same result as the expectation that the surface enhancement between gold-coated nanorods can occur well. Thus, the Raman signal obtained in the urine-diffused region becomes an enhanced signal of the biomarkers located within the nano-sized pores of the SERS substrate.

### 3.2. Raman Measurement and Statistical Analysis

#### 3.2.1. Surface Enhancement Raman Measurement of Nanometric Biomarker

**Figure 4:**
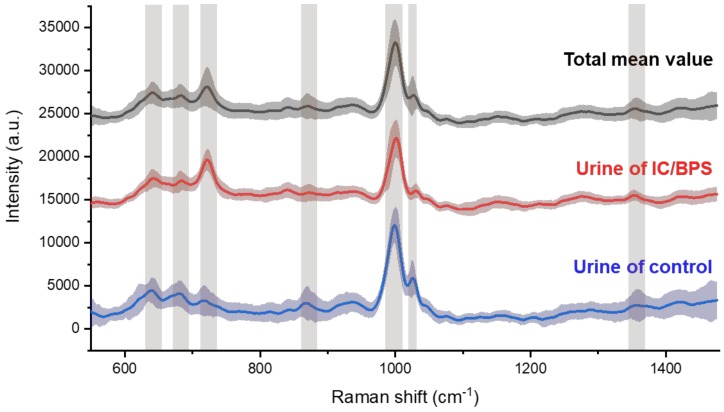
Averaged Raman spectra for all samples (black line), for IC/BPS (blue line), and control (red line) of rat’s urine. Standard deviations are painted around the spectra, and the main peaks marked by bands are due to nanometer-scale bio-markers.

The Raman spectroscopic signals were measured in the diffused region for nanometer biomarker detection, to indicate IC/BPS. By plotting the mean spectral lines and standard deviations together, overall peak position, which are the elements to identify, could be shown (Figure 4). The bands are indicated on the peak, which is the main factor above the graph drawn as the average of the total data. The shaded area with standard deviation means reproducibility, and the area of the painted part will appear in high compression when the deviation of each signal in control and IC/BPS is small and ideally reproducible. The main peaks for the control and IC/BPS samples were observed at 641, 683, 723, 873, 1002, 1030, and 1355 cm^−1^, which corresponded to the C-C twisting mode of tyrosine [27,28], ring breathing of nucleic acids for G [28,29] and A [29,30], C-C stretch of hydroxyproline [27,31], symmetric ring breathing mode [27,28,29,30,31] and C-H in-plane bending mode of phenylalanine [27,31], and CH_3_CH_2_ wagging mode of collagen [27,31], respectively. The peak at 1002 cm^−1^ has a considerably high intensity compared to the rest of the data, which is a notable observation in relation to the literature on other peaks, as these relate to other fields of biology.

#### 3.2.2. Principal Component Analysis

**Figure 5:**
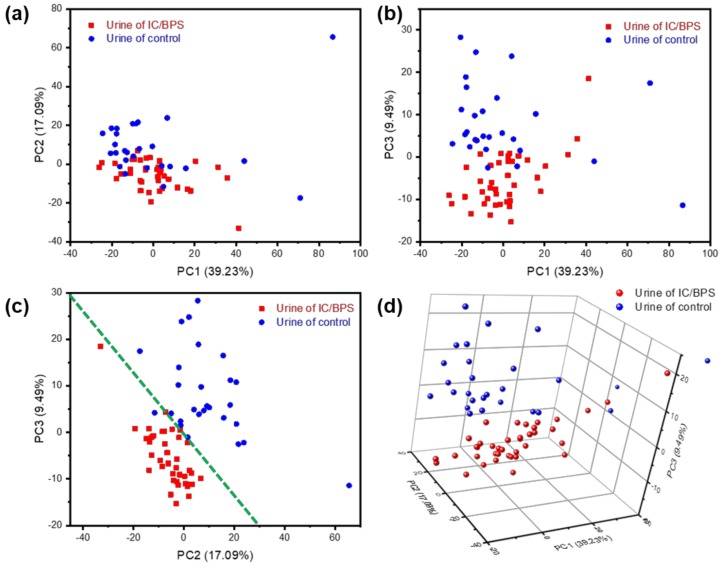
Principal component analysis results for urine of IC/BPS and control sample: (**a**) PC1 (39.23%) vs. PC2 (17.09%); (**b**) PC1 vs. PC3 (9.49%); (**c**) PC2 vs. PC3; (**d**) 3D plot of PC1, PC2, and PC3.

A principal component analysis (PCA) is a standard algorithm which allows the capture of data variability into orthogonal components in highly multivariate data, and is applied in biotechnology such as diagnosis and classification [2,3,4,27,28,29]. As the difference between the IC/BPS and control sample Raman signals seems to be due more to the relative intensities of the peaks than to the peak position shift or peak generation, a clearer separation of the data may be obtained in this application by graphing the data along their principal components. As shown in Figure 5, a clear discrimination and reliable separation between sample groups were observed in the PCA score plot. Although there is a significant amount of random noise, comparing the intensities along the spectral axis, as in Figure 4, shows a variance in the peaks that nearly exceeds the noise threshold. PCA, the correlated change in intensities between comparison groups, can be extracted for a more meaningful signal. Raman shift values were used as the multidimensional variables for PCA, and 67 unique principal components (PCs) were identified from an initial 1024 variables. The first principal component (PC1) explained 39.23% of the variability, the second component (PC2) explained 17.09%, and the third component (PC3) explained 9.49% (Figure 5a–c). Since IC/BPS and control urine were identifiable from voiding patterns, they were marked red and blue in the PCs space and plane, respectively. As shown in Figure 5c, the distribution of data in the PC2 and PC3 planes may be differentiated based on the green dotted line. By plotting PC1, PC2 and PC3 in 3-dimensional space, a clear separation may be seen between the clusters of IC/BPS urine and of the control samples (Figure 5d).

Based on the Raman spectrum measurement and PCA analysis results, it has been experimentally confirmed that it is possible to distinguish between the disease group and normal group using gold-coated ZnO nanorods substrates, which could be applicable in early disease diagnostic sensing chips.

## 4. Conclusions

Interstitial cystitis/bladder pain syndrome (IC/BPS) is a refractory disease accompanied by great pain and discomfort. It can undermine a patient’s quality of life, unless it is detected in the early stages. There are various factors, including red blood cells, epithelial cells, and bacteria, in IC/BPS and in normal urine; finding biomarkers for an early diagnosis among these factors is a major issue. In order to model the urinary disease using an early diagnosis, an IC/BPS animal model was prepared by injecting HCl into the bladder of a rat, and urine was collected a week later. Raman spectroscopy experiments were conducted after growing gold-coated ZnO-nanorods to facilitate size-selective SERS detection. An enhanced Raman measuring approach and PCA analysis confirmed that nano-level biomarkers could distinguish between IC/BPS and normal urine. Nanometer biomarkers are not localized compared to the same volume in urine or blood, unlike cells or bacteria. Further, Raman spectroscopy could provide specific biological information based on their biochemical, physical, and optical responses. Thus, we can confirm that ZnO nanorod-based SERS has ample potential for early disease diagnoses.
